# Will the World Ever Be the Same After COVID-19? Two Lessons from the First Global Crisis of a Digital Age

**DOI:** 10.1007/s40804-020-00194-9

**Published:** 2020-09-10

**Authors:** Mark Fenwick, Joseph A. McCahery, Erik P. M. Vermeulen

**Affiliations:** 1 Kyushu University, Fukuoka, Japan; 2 Tilburg University, Tilburg, The Netherlands; 3 Tilburg University/Philips Lighting, Tilburg, The Netherlands

**Keywords:** COVID-19, Coronavirus, Policy response, Higher-tech education, G18, I1, I2

## Abstract

Coronavirus is the first global crisis of a digital age and the divergence in policy responses reflects the challenge of navigating an unprecedented global situation under conditions of enormous uncertainty. We ask what lessons can be learned from this experience and identify two, both of which push against mainstream interpretations of recent events. First, and contrary to the view that the crisis exposed social media and Big Tech as a source of dangerous misinformation that needs to be regulated more strictly, the paper argues that the less mediated spaces of the Internet—social media and Twitter, in particular—played an essential role in triggering a more effective policy response based around social distancing, lockdown, and containment. Second, and contrary to the view that things will go back to normal once the worst of the crisis has passed, the paper argues that, as a direct result of lockdown, the status quo has been shifted across multiple sectors of the economy. Three examples of this shift are introduced, notably the forced experimentation with digital technologies in education and health, the increased use of remote work in many companies, and a reduction in environmentally harmful behavior and decrease in pollution levels. The long-term effects of this ‘reset’ are impossible to predict, but a quick return to the ‘old normal’ seems unlikely. The paper concludes with the suggestion that this reset has created a unique historical opportunity for the reappraisal of regulatory approaches across multiple domains and exposed the need for regulatory models better aligned to a less mediated, more decentralized world. COVID-19 is a global tragedy, but—given that it has happened—it should be used as a learning experience to re-imagine a better, more socially, and environmentally responsible future.

## Introduction

*This is not a drill.*

*This is not the time to give up.*

*This is not a time for excuses.*

*This is a time for pulling out all the stops.*

No doubt, this sounds dramatic. But, Dr. Tedros Adhanom Ghebreyesus, the Director-General of the World Health Organization (WHO), didn’t think so. He used exactly these words at the media briefing on COVID-19 on March 5, 2020, to encourage national governments and public health organizations to collaborate in the fight against the virus.^1^ In essence, he recognized that only a coordinated approach would help stop the global spread of the disease and avoid catastrophe. To quote him from the conclusion of the same press conference: ‘We’re all in this together, and we can only save lives together.’

No matter what your opinion of the performance of the WHO or the Director-General, he is right about one thing. There was a lack of consensus amongst policymakers regarding how to respond to the unprecedented challenge created by the virus. Governments were not on the same page when it came to the issue of when to respond and what to do.^2^ COVID-19 did not just bring about a rapid rise in policies adopted, but they varied widely from country to country and—in larger, federal countries—from region to region.^3^

Different jurisdictions adopted different ways to count and report cases of infection, different approaches to testing, and recommendations as to what to do if you had a cold or fever.^4^ Similar differences extended to the necessity, scope, and scale of the lockdown, and how best to manage the process of lifting restrictions. Even something as simple as wearing a mask generated disagreement and controversy.^5^ The result? A high degree of confusion that fueled public mistrust of authorities and increased anxiety about the risks.^6^

There are many factors that account for these differences in response. For instance, the level of actual or perceived threat.^7^ Regions that experienced a clear and sudden epidemic tended to implement more stringent measures, as did areas neighboring a severe outbreak. Political considerations also affected policy measures; governments might have decided to downplay the coronavirus threat or underreport the number of cases to avoid criticism, mitigate the adverse economic effects,^8^ or some other reason (for example, to save the Olympics). Many will be tempted to see the failure to cooperate and diversity of response as one element of a broader failure of political leadership.^9^ Clearly, some political leaders have performed better than others. Finally, one should not ignore the cultural differences that impact policy choices.^10^ Deep-rooted traditions in the workplace may make it difficult for people who exhibit the early onset of symptoms to come forward or for lockdowns to be fully effective. This is particularly true in countries with a working culture where a sick day—even a holiday—is frowned upon by peers.

Nevertheless, we explore a different thought, namely that the coronavirus is the first global crisis of a digital age. The divergence in policy responses to the crisis reflects the challenge of navigating an unprecedented situation under conditions of enormous uncertainty.^11^ Although researchers have examined the effectiveness of the relative policy efforts, they have left relatively unexplored the question of the lessons from the COVID-19 pandemic.^12^

In this paper, we start by asking what lessons can be learned from this experience and, then, identify two lessons, both of which push against mainstream interpretations of recent events. First, and contrary to the view that the crisis exposed social media as a source of dangerous misinformation that needs to be regulated more strictly, the paper argues that the less mediated decentralized spaces of the Internet—social media and Twitter, in particular—played an essential role in triggering a more effective policy response based around social distancing, lockdown, and containment.^13^

Second, and contrary to the view that things will go back to normal once the worst of the crisis has passed, the paper argues that, as a direct result of lockdown, the status quo has been shifted across multiple sectors of society. Several examples of this shift are introduced, notably forced adoption of and experimentation with digital technologies in education and health, and a reduction in environmentally harmful behavior and pollution levels. The long-term effects of this ‘reset’ are impossible to predict, but a quick return to the ‘old normal’ seems unlikely.

The paper concludes with the suggestion that this reset has created a unique historical opportunity for the reappraisal of regulatory approaches across multiple domains and exposed the need for regulatory models better aligned to a less mediated, more decentralized world. COVID-19 is a global tragedy, but—given that it has happened—it should be used as a learning experience to re-imagine a better, more socially, and environmentally responsible future.

This paper proceeds as follows. Section 2 explores the pattern of regulatory responses in an environment without information gatekeepers. Section 3 focuses on social distancing and the contribution of the forced adoption of technology to new leadership models and environmental policies. Section 4 concludes.

## Regulatory Responses in a World Without Gatekeepers

To understand the claim that the coronavirus is the first global crisis of a digital age, it is helpful to briefly outline the history of information and the changing role of information intermediaries. Such a history reveals how the digital age breaks with the past, most obviously by undermining the power of traditional gatekeepers.

Consider the world before the invention of the printing press. The primary gatekeeper of information throughout the Middle Ages in Europe was the Catholic Church.^14^ The overwhelming majority of books were written in Latin—the language of the Church—and were reproduced, by hand, by monk copyists—agents of the Church. This difficult and highly skilled work gave the Church an effective monopoly over the content, reproduction, and dissemination of authoritative information. Control of knowledge and opinion was a primary source of church power for several centuries. Access to information was controlled, and even reading and writing were tightly restricted skills.^15^ Secular book production did exist on a limited scale, particularly in university towns from the twelfth century onwards, but the Church remained the dominant source of information.

The emergence of the printing press in the mid-fifteenth century significantly undermined this authority.^16^ For the first time, it allowed competing sources of information and new bodies to emerge that challenged the monopoly of Church teachings and influence. In particular, the printing press decentralized control of information by allowing regional and local authorities to re-interpret the Bible in ways that suited their political interests (think of Martin Luther, instance). The printing press provided an effective means to distribute this information to local populations independently of the distribution channels established and maintained by the Church. Before the invention of the printing press, the number of manuscript books in Europe could be counted in thousands. By 1500, after only around fifty years of printing, there were estimated to be more than nine million books.^17^

The ultimate result of the Guttenberg Revolution—an earlier information revolution—was the emergence of city and nation-states that challenged and, ultimately, replaced the Church as the primary source of political and legal authority in Europe and, as a result of several centuries of colonialism, the rest of the world.^18^

In this way, the printed word (books and newspapers) played a crucial role in transforming the world by democratizing *access* to information. Control over the information that was published provided a new group of ‘Fourth Estate’ gatekeepers (i.e., newspaper and publishing companies) with enormous influence, at least within a specific geographical territory or linguistic community.^19^ Political actors and institutions had to either control these gatekeepers—via state censorship—or work in partnership with them to maintain their legitimacy and authority.

The new importance of consumers, particularly within capitalism, meant that companies confronted a similar need to engage with the information gatekeepers of the Fourth Estate. To distribute information on products and services, companies wanted to get that information to customers. This need led to the emergence and growth of advertising. Advertising provided an additional source of income and economic influence for the gatekeepers.

Finally, scientific knowledge and other forms of expertise needed to pass through intermediaries to find a mass audience. The gatekeepers of the Fourth Estate also took on the critical task of ‘translating’ specialized scientific knowledge into more accessible information that could then be consumed by the population at large. Mass education—and literacy, in particular—gave people the means to acquire the discoveries of modern science and other expert knowledge. However, the consumption of this information was still dependent on intermediaries.

Over the twentieth century, television emerged as a new medium by which information was collected and disseminated within modern societies. However, the basic structure of the information ecosystem was not radically disrupted. Intermediaries—in the form of publishing companies, newspapers, and the new TV stations—were key actors. Their power derived from the control of both the means of information production (most obviously, ownership of printing presses and television studios) *and* the distribution network (e.g., an infrastructure for delivering newspapers, a chain of bookshops, or a broadcasting network). This power meant that—in liberal democracies, at least—a relationship of co-dependence existed between information intermediaries and other influential groups within society, most obviously political actors, large corporations, and scientific and educational institutions.

The emergence of the Internet, however, changed everything.^20^ Particularly post-2007, with the introduction and mass dissemination of smartphones, information can now be published instantly by anyone to a potentially global audience. There are approximately 5 billion people that now have mobile devices and over half of these have access to a smartphone.^21^ Digital technologies have already connected almost everyone, and it won’t be long before they have linked most of the rest.

When most of the global population owns a powerful mobile device that connects them to the Internet, we have moved into a new information age. Talk of a ‘revolution’ is justified. In our lifetimes, we have witnessed an event equivalent in significance to the invention of the printing press. We can refer to this new age as a networked age, the Internet age, or a digital age—the label doesn’t matter. However, the combination of a global communications network and the mass availability of powerful digital devices hooked up to that network has transformed the information ecosystem of our societies and culture.

Most significantly in this context, the combination of ‘Internet-plus-smartphones’ poses an existential threat to the power of the ‘Fourth Estate’ as it gives anyone the means to communicate their message-information directly to the whole world without the need for any intermediaries. We have left behind the ‘closed’ world of traditional gatekeepers in which only a small number of elite voices were heard.

Mark Zuckerberg described the radical disruption that the Internet and social media has created in his description of the new ‘Fifth Estate’ in an October 2019 address at Georgetown University:

People having the power to express themselves at scale is a new kind of force in the world—a Fifth Estate alongside the other power structures of society. People no longer have to rely on traditional gatekeepers in politics or media to make their voices heard, and that has important consequences. I understand the concerns about how tech platforms have centralized power, but I actually believe the much bigger story is how much these platforms have decentralized power by putting it directly into people’s hands. It’s part of this amazing expansion of voice through law, culture and technology. So, giving people a voice and broader inclusion go hand in hand, and the trend has been towards greater voice over time.^22^

Set aside skepticism about Zuckerberg’s motives and recognize the critical shift that occurs when ‘people no longer have to rely on traditional gatekeepers in politics or media to make their voices heard’. While real disagreement persists about the influence of Facebook or big tech companies,^23^ this constitutes a truly historical shift in the structure of the information ecosystem, and societies and economies more generally.

Unsurprisingly, incumbent gatekeepers—the publishing companies, the newspapers, and the TV companies—were quick to recognize this rival source of information as a threat and have invested heavily in seeking to discredit these emerging rivals. Somewhat predictably, incumbents tend to focus on the downside that this development has created. It is not difficult to find stories in the ‘old media’ on foreign state interference using social media and other online platforms to influence, for example, the 2016 US presidential election or the Brexit referendum.

This attitude is hardly surprising. After all, the whole model of information gatekeepers is premised on the idea that *they*—the centralized incumbents—are the only source of ‘good’ information within society and that people are willing to pay for information curated by a trusted source. If an alternative—and free—source of trusted information is available, then the business model of the Fourth Estate is greatly undermined.

To be clear, more information *in total* is available on the Internet and, therefore, there is inevitably more ‘bad’ information available. If you want to find bad information online, it is not difficult. However, the thought we want to pursue here is that there is also more good information and this information can play an important function in a crisis.

Here—and for no particular reason—we will refer to this new age as a ‘digital age’, and our suggestion is the coronavirus is the first global crisis of a digital age (post-2007).

A common media narrative throughout the crisis is that social media has been a negative influence. There have been numerous articles in the traditional media condemning the role of social media. Take this article from the Guardian newspaper, which is worth quoting at length:

[M]isinformation continues to adapt and spread, largely on social media. Research by Oxford’s Reuters Institute looking at the spread of 225 false or misleading claims about coronavirus found 88% of the claims had appeared on social media platforms, compared with 9% on television or 8% in news outlets. Nearly 30% of US adults believe Covid-19 was developed in a lab, according to a survey by Pew Research Center. A conspiracy theory falsely linking 5G to the coronavirus pandemic has led to real-world consequences, including threats and harassment against telecom engineers and petrol bomb attacks on telephone poles. Carl Bergstrom, a University of Washington professor of biology who also studies and has written a book about misinformation, says the efforts of the social media companies are too little, too late. ‘They’ve built this whole ecosystem that is all about engagement, allows viral spread, and hasn’t ever put any currency on accuracy’, he said. ‘Now all of a sudden, we have a serious global crisis, and they want to put some Band-Aids on it. It’s better than not acting, but praising them for doing it is like praising Philip Morris for putting filters on cigarettes’ […] ‘We planned for years for this pandemic, but we never realised that we would be fighting a war on two fronts’, said Bergstrom. ‘One against the pandemic, and one against all the disinformation and hate and fear that is being amped up and enflamed by political opportunists.’^24^

Similar stories can be found in other newspapers.^25^ Again, it is hardly surprising that traditional gatekeepers have used the crisis as an opportunity to attack rival sources of information. And, in an information-rich digital age, there will be more inaccurate information and more extreme opinions. That is an inevitable consequence of removing the need for gatekeepers in the information ecosystem of a digital age.

However, there is another side to this story, which is not reported as frequently by the traditional gatekeepers. For sure, there is more misinformation, but there is also a lot more ‘good’ information, and much of this good information was not previously disseminated or—if it was—it was heavily filtered by intermediaries. The importance of this ‘good’ information in changing the narrative and pushing political actors into more effective action should not be obscured by the existence of misinformation, conspiracy theories, or extremist opinion.

Tech analyst, Ben Thompson describes the more positive effects of the new media as follows:

From January on there has been extensive information about COVID-19 shared on Twitter, in particular, including supporting blog posts, and links to medical papers published at astounding speed, often in defiance of traditional media. In addition, multiple experts, including epidemiologists and public health officials, have been offering up their opinions directly. Moreover, particularly in the last several weeks, that burgeoning network has been sounding the alarm about the crisis hitting the US. Indeed, it is only because of Twitter that we knew that the crisis had long since started.^26^

In short, a world without gatekeepers has a significant upside, as well as a downside. And this upside has played a crucial role in exposing, criticizing, and correcting regulatory responses.

In considering the positive influence of social media, it is helpful to differentiate three functions that it can perform and illustrate with recent examples.

Firstly, social media can help correct an influential narrative that has gained traction in the mainstream media and is framing and influencing policy choices in a negative manner (the ‘corrective function’).

An example of this can be seen in the US. For much of the period between January and March, there was a widespread narrative—perpetuated by an unlikely alliance of conservative politicians and the liberal media—that we should be more worried more about influenza than coronavirus. The New York Times, for instance, published several articles comparing the coronavirus to the flu and warned that overreaction would be worse than the pandemic.^27^ The author of one such article later admitted he had been wrong, but that it was a ‘black swan’ event, i.e., the seriousness of a new strand of coronavirus could not have been reasonably foreseen at the time of writing.^28^ More controversially, another piece, on February 5, written by the paper’s tourism-industry correspondent, claimed that travel bans were unjust, ineffective, and racist. Primarily since they were not issued for regions affected by influenza, but only areas affected by the coronavirus.^29^

Social media played an important role in destabilizing this ‘it’s no worse than the flu’ narrative and prompting a more serious regulatory attitude. A particularly notable moment in this shift was a Twitter thread on March 1, from Trevor Bedford, a member of the Seattle Flu Study team.^30^ The Seattle Study team examined the genome of a local case and concluded that there had been transmission in Washington State for at least six weeks and that they were already facing a substantial outbreak. This information—from an ‘old-world’ expert, disseminated via Twitter—played a pivotal role in triggering social distancing on the West Coast. Many companies introduced remote work, travel decreased rapidly, and conferences and other events were canceled. This bottom-up action occurred, while centralized authorities and information intermediaries failed to provide appropriate leadership.

Second, social media can hold government to account and force government to disclose the basis of their decisions and/or make to better decisions (the ‘accountability function’).

An example of this can be seen in the UK. At least initially, the Conservative government of Boris Johnson favored a so-called ‘herd immunity’ approach in which the virus would be allowed to spread throughout the population in order to generate community immunity.^31^ The government obscured this point and did not publish the data or models on which such an approach was based. This policy was first exposed and then heavily criticized for its inhumanity—particularly on Twitter—and was quickly changed to the policy of containment and lockdown favored elsewhere.

This shift of direction was marked by two crucial factors. Firstly, hundreds of scientists signed a letter that circulated online.^32^ And epidemiologists at Imperial College (a part of the University of London) published a study—based on data gathered from Italy—that aimed to convince the UK government to impose a suppression strategy to contain the COVID-19 outbreak.^33^ Broadly speaking, bottom up action, and information distributed online played a crucial role in holding government to account and prompted a policy U-turn. The mainstream media picked up on these stories and played a crucial role, but they did not originate these actions and we cannot be sure that they would have developed the force that they did in the absence of a large social media response.

Finally, social media can provide a forum for all politicians, but particular more marginal political figures to communicate directly with the people, which creates political pressure on mainstream political actors to make better decisions (the ‘platform function’).

An example of this can be seen in Japan. A striking feature of the Japanese experience is the degree to which local politicians, especially prefectural governors, have driven the policy debate and national leaders—particularly PM Abe—have been reduced to the role of playing catch up.

Significantly, the more effective use of new media has been central to this success. For example, Tokyo Governor, Yuriko Koike, won a lot of praise for her interview with YouTuber Hikakin.^34^ He is well-known for strange antics on his channel, but this interview ended up being an informative discussion that offered genuine insight into COVID-19 and how to curb its spread in Japan. Similar use of social media to engage with broader constituencies has also characterized another political leader to have benefited from the crisis, Osaka Prefectural Governor, Hirfumi Yoshimura. The effective use of social media as a platform along with traditional gatekeepers (TV and newspapers) allowed local politicians to take on a national leadership role that drove the response of central government. And, apparently, the success of this use of social media triggered PM Abe to respond with his own online campaign—which proved much less well-judged.^35^

The crucial point is that crisis communication requires a delicate balancing of information and mood. Social media provides a platform for both political leaders and other voices (including more expert voices) to communicate directly to the people and with sections of the population that may not consume information from traditional gatekeepers. This increases participation in political debate and policy making process broadly defined. More citizen engagement creates more dialogue, and more trust. So, when Governor Koike later requested Tokyo residents to ‘Stay Home’ her message was well-received and effective, partly as a result of her strategy of communicating across multiple platforms.

In sum, in the case of COVID-19, there are many examples of how information published online helped shift the policy making process in a positive direction. In this way, social media contributes to a culture in which people demand to know how decisions are made.

There are risks that these functions can be abused. But, we should have more faith in the ability of people to distinguish between reliable and unreliable information, and information that is simply impossible or difficult to judge. Research suggests that young people (‘digital natives’), in particular, are adept at reading information online and recognize the importance of individual verification.^36^ People don’t simply believe everything that they read online, but take a more subtle approach that involves balancing and comparing any new information with existing knowledge and beliefs. Another study found that social media resulted in more diverse viewpoints relative to offline news consumption.^37^ Again, this is not to say that everything is perfect, either in terms of the coronavirus or unmediated information, in general. However, it does suggest that a simple critique of the new media is misplaced and—that contrary to the narrative perpetuated by traditional gatekeepers—the new media has functioned well in the current crisis in the three senses described above, and—in many cases—better than the conventional information gatekeepers.

## Social Distancing, Lockdown and the ‘Reset’ of Reality

So far, this paper has addressed the effects of social media on mainstream media’s framing and influencing of government COVID-19 strategies. Our analysis also considered the practice of social media to hold governments to account, and force policymakers to disclose the basis of their decisions. In this section, we turn to address the near and mid-term impact of social distancing and enforced lockdown measures on the expanding role of technology in education and work, alternative leadership and environmental strategies. Before studying the factors leading to policy change, we need to address a preliminary question: When should we expect that the regulatory status quo is effectively destabilized, and a clear direction in policy has shifted?^38^

Almost twenty years ago, Ronald Gilson observed that defending the status quo is always easier than achieving change. Gilson argued that ‘accomplishing change is more complicated than merely having to protect the status quo’.^39^ By contrast, he also noted that an unexpected event, triggering a change to the status quo, can result in a new normal that then, itself, becomes difficult to dislodge.^40^ He referred to an example:

An example from San Francisco, where I live, illustrates the point. For years, many residents sought to have the Embarcadero Freeway, a two-level concrete abomination that separated downtown San Francisco from the waterfront, demolished. While this group was likely a majority, a concentrated minority whose businesses the freeway benefited was successful in blocking demolition. The 1989 earthquake, operating as a mechanism of natural urban renewal, inflicted sufficient damage that the freeway had to be torn down. Thereafter, the freeway proponents were unable to muster the political influence to have it rebuilt.^41^

When the 1989 Loma Prieta earthquake inflicted significant damage, opponents of the freeway were able to push for the complete removal of the damaged structure. Although the ‘concentrated minority’ would suffer a dramatic decline in business, once the destruction had become a social fact, the freeway was torn down and never rebuilt. The earthquake radically changed the status quo—it reset reality—and that shift transformed the terms of the debate and the policy choices, and affected the ultimate outcome.^42^

Of course, one should be cautious to compare an earthquake with the outbreak of the coronavirus. However, similar to what happened in California in 1989, the coronavirus is changing the status quo in many areas, as a direct result of the effects of a policy of containment based around social distancing and lockdown. The medium to long-term effects of this reset are uncertain and difficult to predict, nevertheless, it should not be assumed that things will go back to normal anytime soon.^43^ As Gilson observed, any change in the status quo has important effects. And the long-term repercussions are not always predictable. Likewise, we will consider the following examples of how the lockdown has changed the status quo.

### The Forced Adoption of Technology

Let’s start with the first example, The forced adoption of new technologies in recent months means that people have been compelled to use technologies that they might never have used and this experience, happening on a large scale, redefines the status quo.

Venture capitalist and tech analyst Benedict Evans uses the concept of the ‘forced experiment’ to describe how engagement with a new technology changes how we work:

Every time we get a new kind of tool, we start by making the new thing fit the existing ways that we work, but then, over time, we change the work to fit the new tool. You’re used to making your metrics dashboard in PowerPoint, and the cloud comes along and you can make it in Google Docs and everyone always has the latest version. But one day, you realise that the dashboard could be generated automatically and be a live webpage, and no-one needs to make those slides at all. Today, sometimes doing the meeting as a video call is a poor substitute for human interaction, but sometimes it’s like putting slides in the cloud. I don’t think we know which is right now, but we’re going through a vast, forced public experiment to find out which bits of human psychology will align with which kinds of tool, just as we did with SMS, email or indeed phone calls in previous generations.^44^

Adoption results in learning, which triggers experimentation and, ultimately, adaptation and evolution.^45^ And, this process is continuous and irreversible. There is rarely any ‘going back’ once the status quo has shifted. At least, any ‘return’ to the past is complicated by the new experience that has occurred in the interim. We may not know the future, but we know it will be different from the past.

Yet despite these obvious benefits from breaking with routine, studies have found that many people may not be particularly well-prepared to experiment. One study of the strike that affected the London underground network between 5 and 6 February 2014, found not only that certain commuters experimented and explored new routes on those days, but were able to take advantage of the useful information produced to switch to more efficient routes post-strike.^46^

A shift in the status quo of this kind can be seen, most obviously, in two sectors where there has long been talk of ‘going digital’ but very little changed occurred: education^47^ and health. Both sectors have now entered a process of forced adoption and experimentation with technology as a result of lockdown and social distancing.

In the past, a significant problem for any young company looking to operate in the education or health sectors was the challenge of getting users to adopt a disruptive new product. This is a problem for any disruptive firm in any sector of the economy, but there are a number of sector specific issues that have made this challenge insurmountable in education and health.

Most obviously, the users of education and health products—schools, universities, hospitals—are typically slow to change anything as a result of bureaucratic-style organization and complex regulatory environment. Also, the people who *use* the products in these sectors (teachers and students, doctors and patients) are usually not the people making the choice about service providers (typically this would be education and hospital administrators). Cost concerns often triumph over real world usability as the primary metrics driving product or service providers. A fear of something going wrong (and the inevitable criticism that would follow) creates an understandable caution and a tendency to stick with what you know. Finally, the necessity of concluding long term contracts in order to have stability creates ‘lock in’ effects that make it difficult to switch to new service providers that would require additional training in the absence of a strong need or justification.

The result? In the past, it was not enough for disruptive education or health startups to have a great product or service. They faced enormous transaction costs in getting those great products or services to users at a scale necessary to grow a business.

Coronavirus has changed everything. Take education.^48^ By March 4, 2020, UNESCO announced that thirteen countries implemented school closures, affecting 290 million students globally.^49^ The risks of such closures are obvious: lost education time, disillusionment and anxiety, higher dropout rates, and other social costs (family troubles and anti-social behavior). The solution? Remote learning.^50^ Governments and schools have no other choice than to promote and facilitate online classes.

There are plenty of education startups that can help pave the way to develop distance learning environments.^51^ The most familiar type is the massive open online courses (MOOCs) and other forms of distance learning. In their first decade the rise of online education has contributed to new learning experiences, content and teaching methods. Indeed, MOOCs have created access and engagement with students and communities. At the same time, the MOOC format has brought up concerns about quality of learning, pedagogy and the limited success with accreditation.^52^ That said, it has always been difficult to change the status quo of in-class learning. But, the urgency of the current situation (lockdown and the need for remote work/learning) has changed the decision-making calculus of schools and school administrators and created a new status quo in which remote learning has become standard. Changes that might have taken decades occurred in days.^53^ Not every change will last once schools reopen, but forced adoption of technologies and the resulting experimentation that has ensued will create a new post-corona reality that will not be the same as before.^54^

There will be losers as a consequence of this change. Old world retail—already under threat from ecommerce—looks especially vulnerable to a new world of social distancing. Equally, there are well-known examples of technology companies that benefit from a change in the status quo. Think about companies that build an ecosystems around GPS based proximity monitoring. These emerging tech solutions are being employed to prevent the further spread of the virus. Many of these technologies weren’t previously accepted by society, as they raise privacy concerns, but now they have a new meaning when they help doctors with diagnosing coronavirus infections.^55^

We shouldn’t presume that we can predict the future, but nor should we assume that this experience will be quickly forgotten. The only thing we can predict is that technology has played a central role in navigating the crisis and that, moving forward, it will be harder to resist the case for further expanding the role of technology in an education or health context.

### Remote Work & the Changing Meaning of Leadership

We turn now to remote work and the changed meaning of leadership. Many companies have been compelled to introduce remote work as a result of lockdown. Working from home remain controversial issues in most companies. Deep-rooted work cultures often mean that distance working is perceived something bad and that—as a consequence—working from home should remain a short-term exception.

However, forced adoption and experimentation with remote work has now occurred and this resets reality in some less obvious ways. For example, traditional, hierarchical leadership styles don’t work in a ‘working from home’ environment. Command and control isn’t useful in a digital environment, where more distance inevitably means less control. That seems unavoidable. Better companies and leaders recognize this and adapt to the new normal by adopting leadership style—what we will call ‘digital leadership’.

In describing this new style of leadership, we are not referring to the many personal video messages and open letters that have been distributed by corporate leaders during the crisis. Such messages deliver bad news, express dubious emotions, and outline how company leaders have made a ‘sacrifice’ by giving up a percentage of their salary or making donations to charities. Instead, we are referring to creating an open and inclusive working environment that enables every office worker to become a leader for themselves.

Partly, this is a question of trust. Digital leaders understand that they must trust their workforce to exercise the new ‘freedom’ of remote work in a responsible manner. Leaders also become more approachable. Back in the office, they were usually surrounded by the same people over and over again (the ‘blockers’ who kept old world leaders insulated and at a distance from other workers). Leaders existed in an ‘echo chamber’ in which their own ideas were constantly repeated back to them. Now, we see more personal (not pre-recorded) messages that give directions, explanations, and show empathy and understanding^56^ (we are all in this together, and together we have to find adequate solutions).^57^ The best leaders open up, invite questions, have open virtual coffee/tea hours where other workers can ask questions and pose ideas. This allows them to find the people who matter (despite the hierarchy and seniority). These are creative minds who can help the company succeed. In this way, digital leaders ensure that the organization has the necessary ‘shock absorbers’ for a business to deal with risks and uncertainties and, second, they help accelerate the future.

This approach becomes crucial in experimenting and adapting to a post-coronavirus world.^58^ Changes are accelerating and do not always follow pre-defined trajectories. The winning companies of the future will be the ones that define the ‘next normal’ themselves.^59^

For example, the virtual environments of remote work also allow us to collaborate more and faster with my colleagues. There is no need to make appointments. We don’t have to be stuck in traffic and think about what to wear. Authenticity rules. Children on laps, pets in the background, and unshaved men at video conferences don’t matter anymore. In informal settings (with the added comfort of being at home), it is all of a sudden so much easier to connect, interact, exchange ideas, and be innovative. And never forget. The office was never that great anyway. The gossip and back-biting, the nay-sayers and free-riders. The processes, procedures and politics that were a constant obstacle to ‘getting good things done’. Ironically, social distancing has brought us closer together and created a better environment for serendipity than the open spaces or bathrooms of the modern office.

Identifying how things are changing and adapting to that change becomes vital. This means living without a script and maintaining a willingness to be flexible and changing your mind. Those moments of transition. Originality, vision, and craft and not repetition and routine. Making markets not following them. Unbounded by geographic constraints, remote work offers the possibility of a future orientation and developing a culture that facilitates innovation.

### Greener Cities

Our final example of the shift in the status quo triggered by the crisis is not related to the adoption of new technology, but the environment. Amongst the most striking images of the crisis are satellite images that show the indirect impact of the coronavirus on the environment. The air above affected areas has become much clearer as pollution levels go down as a result of the effects of lockdown, i.e., factory closures and travel restrictions. For example, carbon dioxide emissions fell dramatically as a result of lockdowns. Daily emissions of the greenhouse gas dropped 17% by early April compared with 2019 levels, according to a study of global carbon output published in mid-May 2020.^60^

In the same way that technologies have struggled to break through, environmental concerns have often struggled to make themselves heard in a world where economic growth is prioritized. Nevertheless, lockdown and the need for social distancing have given new impetus to more environmentally-oriented policies.^61^ It should come as no surprise that empirical studies show that companies have raised their ESG scores as a measure of a responsible business orientation and commitment to stakeholders.^62^ Furthermore, there is substantial investor demand for companies to incorporate ESG into their strategic planning. As such, it highlights the fact that investors, as is evident in the growth rate of sustainable investments under management, are willing to accept lower risk-adjusted returns so long as their investments exhibit high ESG qualities.^63^ Even amid the COVID-19 crisis, ESG funds outperformed the wider market. The end result is that a company’s ESG score is perhaps one of the most important factors determining their performance.^64^

Similarly, the COVID-19 crisis had an immediate impact on the travel patterns and public transport sector.^65^ Given the fact that transport systems cannot operate at anything like full capacity for the foreseeable future, many cities are taking measures to facilitate alternatives.^66^ If there is a massive switch to car use, the resulting gridlock and pollution will create additional problems, so these alternatives involve facilitating walking and cycling. For example, many cities have already announced plans for new bike lanes (e.g., London and Milan) and pedestrianizing neighborhoods (e.g., New York). These measure are clearly designed to make it easier to maintain social distance in urban spaces, but a positive secondary effect of such measures it that they don’t increase pollution and nudge people into adopting more environmentally friendly forms of behavior.^67^

For example: In London, the Mayor announced plans to make much of the city car-free with access limited to buses, pedestrians and cyclists.^68^ Mexico City is planning to introduce up to 80 miles of temporary bike lanes.^69^ Milan has introduced one of Europe’s most ambitious cycling and walking schemes, with 22 miles of streets to be transformed over the summer.^70^ New York has plans to open up a new network of streets for ‘socially responsible recreation’ during the COVID-19 crisis, with a focus on areas with the most need.^71^ In Paris, the mayor has allocated €300 m for a network of cycle lanes, many of which will follow existing metro lines, to offer an alternative to public transport.^72^

Rachel Aldred, an expert on urban transport systems notes the critical importance of the next few months as we emerge from the immediate threat of COVID-19:

This is a really important moment. There is the potential to lock in the reduction in air pollution we have seen over the past weeks if we get this right, but as people begin to go back to work and can’t or don’t feel safe using public transport, there is the potential to instead lock in a huge increase in car use and pollution.^73^

Many of these measures were introduced as short term responses to an ongoing threat. But with the Embarcadero Freeway story in mind, it isn’t immediately obvious that industry and transportation systems will easily return to a pre-coronavirus form after the high alert phase of the pandemic has passed. It seems likely that this is how cities are going to function for the next couple of years, and that they can easily become permanent, especially if we have a ‘green economic recovery’. However, such an outcome is not inevitable and—as the quote from Aldred makes clear—it is possible to envisage an alternative scenario in which social distancing takes the form of increased car use and pollution.

## Conclusion

The Director-General of the World Health Organization call for a collective and coordinated response to COVID-19. Of course, governments should set political rivalries and self-interest aside and work together to fight the coronavirus. That much seem obvious. And, anyway, trying to bury information about coronavirus infections is wishful thinking in an age of social media platforms that allow anyone to publish (almost) anything instantly. The crisis has revealed unmediated sources of information to be enormously important in getting political actors to make better decisions.

The coronavirus is the first pandemic of a digital age. Technology has made our world flatter, more transparent, and more decentralized. But, many sectors of the economy and society have resisted the process of ‘going digital’. Vested interests and traditional conservative organizational structures have insulated large parts of society from enjoying the full benefits of digitalization. COVID-19 has forced adoption of new digital technologies and many aspects of the experimentation and learning that the crisis has trigged will prove hard to dislodge.

The benefits of these emerging technologies are clear in the current health crisis and will not be easily forgotten afterwards. New ecosystems will quickly move to other use-cases in other industries and businesses. Technologies that were already near mainstream pre-corona will find it easier to enter traditionally hard to enter markets. The fact that the technologies are being used to make outbreak predictions and search for treatments will also make their use, post-corona, stronger.

Ultimately, of course, businesses, governments and regulators need to understand the full impact of the pandemic and the new reality that has emerged. Only then will we be able to adequately respond to this crisis (as well as future outbreaks), while at the same time identifying regulatory structures for a greener, more sustainable, tech-oriented world. COVID-19 is a tragedy but—given that it has happened—we must use it as a learning experience and an opportunity to re-imagine the future.

## Footnotes

1 WHO (2020).

2 Oxford University (2020), the Oxford COVID-19 Government Response Tracker (OxCGRT).

3 See Weible et al. (2020).

4 OECD (2020a).

5 On the mixed messages related to masks, see Tufekci (2020a).

6 See, e.g., Jaiswal et al. (2020).

7 See, e.g., Fetzer (2020).

8 See e.g., Kager and Rajan (2020).

9 See Tufekci (2020b).

10 See e.g., Harford (2020).

11 See OECD (2020b).

12 For a discussion of the legal lessons, see Johnson and Bailey (2020).

13 For a discussion of the role of social media see Donovan (2020).

14 Concede this is a European perspective. Paper was invented in China in the second century. Cf. Edgren (2010).

15 Clanchy (2010).

16 See e.g., Eisenstein (1979), Febvre and Martin (2010).

17 See Artz (1980).

18 See e.g., Rajan (2019).

19 Carlyle (1841) (noting that ‘Burke said there were Three Estates in Parliament; but, in the Reporters’ Gallery yonder, there sat a Fourth Estate more important far than they all’), p 392.

20 See Carr (2004) (quoting Cisco CEO John Chambers’ speech to IT managers, in February 2001, in which he observed that: ‘the Internet changes everything…’), p. 121.

21 Silver (2019).

22 Romm (2019).

23 See Philippon (2019).

24 Wong (2020). See also Philips (2020).

25 See e.g., Frenkel (2020).

26 Stratechery (2020).

27 Manjoo (2020a).

28 Manjoo (2020b).

29 Spinks (2020).

30 Bedford (2020).

31 On change of direction, see Young (2020).

32 Arrowsmith (2020).

33 Ferguson et al. (2020).

34 ItsYeboi (2018).

35 Johnson (2020).

36 Flamingo/Reuters Institute Oxford University (2020).

37 Reuters Institute (2020).

38 See e.g., Adam et al. (2015).

39 Gilson (2001).

40 Ibid.

41 Ibid., p 354, fn. 87.

42 See Birkland (1997).

43 See Bol (2020).

44 Evans (2020).

45 See e.g., King (2020).

46 See Larcom (2015).

47 See e.g., Govindarajan and Srivasta (2020).

48 See Marjanovic (2020).

49 UNESCO (2020).

50 See Chetty (2020).

51 See e.g., Tabarrok (2012).

52 See e.g., UK Department for Business Innovation & Skills (2013).

53 See World Economic Forum (2020).

54 See e.g., Harris (2020).

55 See Mello and Wang (2020).

56 See Neeley (2015).

57 See Joy (2020).

58 See e.g., Robison (2020).

59 See e.g., Deloitte (2020).

60 Le Quéré (2020).

61 See Albuquerque (2020).

62 See KPMP (2020).

63 See JP Morgan (2020).

64 See Friede et al. (2015).

65 See Sung and Monschauer (2020).

66 See MSCI (2020).

67 Taylor and Laville (2020).

68 Ibid.

69 Ibid.

70 Ibid.

71 See Kuntzman (2020).

72 See Reid (2020).

73 University of Westminster (2020).
